# Supplementary material for: Detection of c.375A>G, c.385A>T, c.571C>T, and sedel2 of FUT2 via Real-Time PCR in a Single Tube
Source: Diagnostics (Basel). 2023 Jun 10;13(12):2022. doi: 10.3390/diagnostics13122022 (PMC10297093; doi:10.3390/diagnostics13122022)

(Reverse sequence)

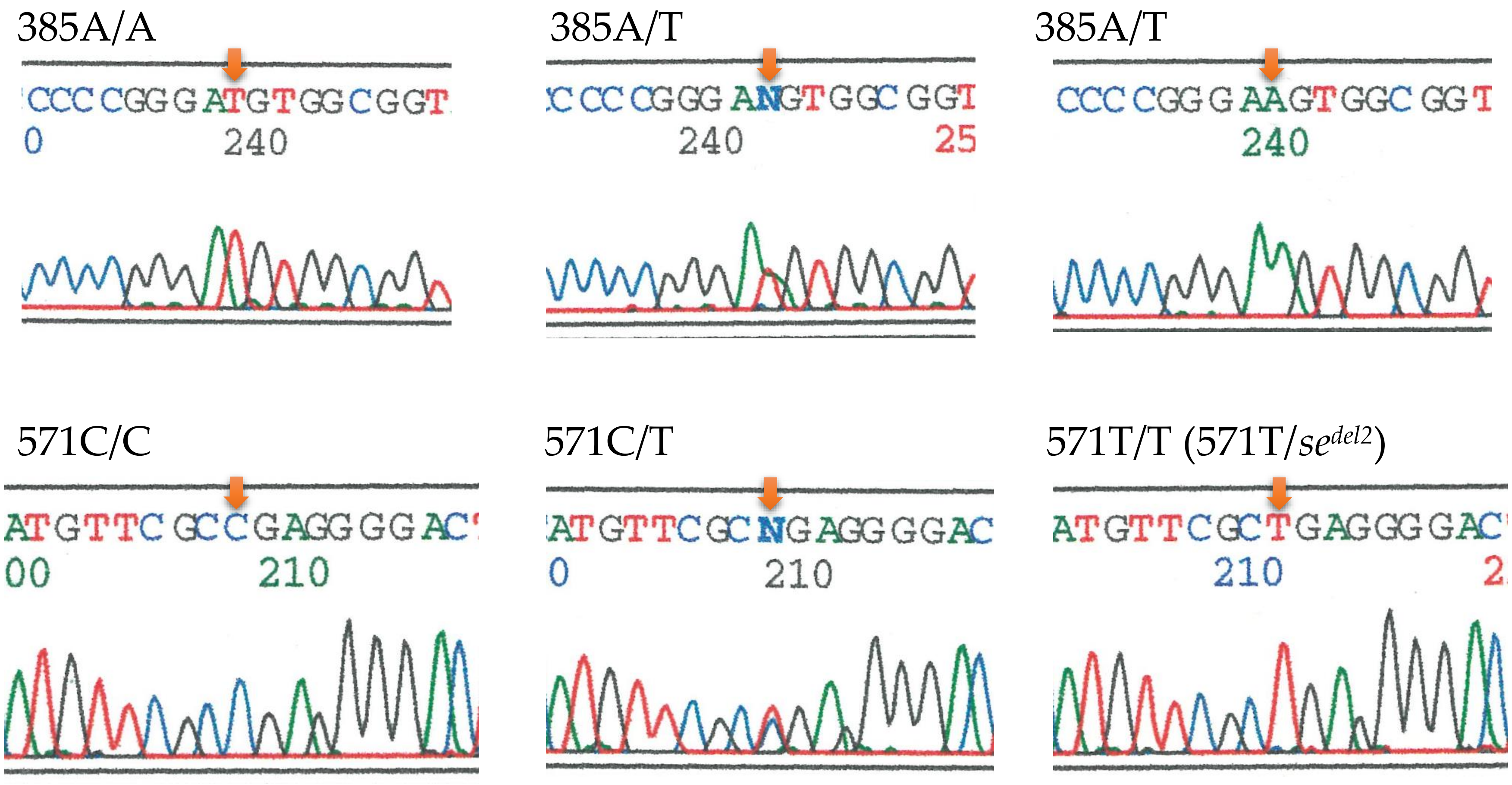

Supplement: Supplementary file 1 [file diagnostics-13-02022-s001.zip › diagnostics-2367033-supplementary.pdf]
